# Factors affecting social phobia among Chinese college students in the context of COVID-19 pandemic: a cross-sectional study

**DOI:** 10.1038/s41598-023-48225-y

**Published:** 2023-11-28

**Authors:** Hai Lin, Ziming Yang, Shanshan Huo, Caixia Su, Zhongsong Zhang, Yingting Rao, Hui Yin

**Affiliations:** 1 Institute of Reproductive and Child Health, Peking University/ Key Laboratory of Reproductive Health, National Health Commission of the People′s Republic of China, Beijing, 100191 China; 2 Department of Epidemiology and Biostatistics, School of Public Health, Peking University, Beijing, 100191 China; 3 Key Laboratory of Epidemiology of Major Diseases (Peking University), Ministry of Education, Beijing, 100191 China; 4 Department of Health Policy and Management, School of Public Health, Peking University, Beijing, 100191 China; 5 School of Public Health, Peking University, Beijing, 100191 China; 6 Institute of Global Health, Peking University, Beijing, 100191 China

**Keywords:** Epidemiology, Risk factors, Psychology

## Abstract

Social phobia (SP) refers to excessive anxiety about social interactions. College students, with their exposure to academic, familial, and job-related pressures, are an ideal population for early screening and intervention of social phobia. Additionally, COVID-19 prevention measures including keeping social distance may further impact social phobia. This study aims to investigate the influencing factors of social phobia among Chinese college students and to tentatively explore the impact of COVID-19 prevention measures on social phobia. Respondents were recruited through Chinese Internet social platforms for an online survey. College students’ social phobia scores in pre- and early-COVID-19 periods were measured using Peters' short form of the Social Interaction Anxiety Scale and Social Phobia Scale (SIAS-6/SPS-6). Demographic information, family information, social relations, self-evaluation, and subjective feelings regarding the impact of COVID-19 preventive measures on social phobia were collected. A multivariable logistic regression model was used to analyze the influencing factors. A total of 1859 valid questionnaires were collected, revealing that the social phobia scores increased from 12.3 ± 11.9 to 13.4 ± 11.9 between pre- and early-COVID-19 periods, with an increase of 1.0 ± 6.4 (p < 0.001). Low GPA rank, mobile phone dependence, distant family relationships, indulgent parents, childhood adversity, and childhood bullying were risk factors for social phobia among Chinese college students. Female gender, being a senior university student or postgraduate, satisfaction with physical appearance, self-reported good mental health and high level of interpersonal trust were protective factors for social phobia. Although most respondents believed that COVID-19 prevention measures (e.g., mask wearing and social distancing rules) reduced their social phobia, these measures were not significantly associated with social phobia levels in the multivariable analyses. In conclusion, Chinese college students’ social phobia was widely influenced by diverse factors and warrants increased attention, with early intervention aimed at high-risk individuals being crucial for their mental health. Additional research is necessary to understand the impact of COVID-19 preventive measures on social phobia among college students.

## Introduction

Social phobia (SP) refers to excessive fear of social situations, fear of attracting attention to oneself in front of others or the public, abnormal trepidation, anxiety, and avoidance of new circumstances or unfamiliar objects^1,2^. SP is a negative psychological emotion that begins in adolescence and is most common in adolescents^3^. It is often accompanied by excessive discomfort, negative reflections, and somatic symptoms (such as blushing, trembling, and sweating that happen before, during, and after social activities), seriously affecting the study and everyday life of young people^4^. Given the early onset and persistence of social phobia^5^, college students are an ideal group for early screening of social phobia as they are new to society and are under pressure from academics, family, job hunting, and courtship^6^. By focusing on the social phobia of college students and the factors influencing them, we can identify groups that are vulnerable to social phobia and provide early intervention and care.

According to previous studies, the factors affecting college students’ social phobia could be divided into three aspects of individual, family, and social relations. Individual factors included physiological reasons, nationality, educational level, childhood adverse experiences, personality, self-efficacy, self-esteem, academic performance, and satisfaction with their appearance^7–10^. Family factors included monthly family income, parenting style, family structure, family relationship, only children or not, rural or urban residence^7,8,11–13^. Social relation factors included school environment, whether they have served as student leaders, social support system^7,8,11–16^. Developed countries have carried out extensive research on the influencing factors of young people's social phobia^17,18^. However, the research on social phobia in China occurred late such a concept was not proposed until 1993^19^. Previous studies on the influencing factors of Chinese college students' social phobia often led to unstable or even contradictory results^7^ because the objects were collected from single regions^11,13^, colleges with specific majors^8,12^, or the sample size was relatively small^20,21^. Many studies focused on a certain province or city, and nationwide studies were rare^7,8,11–13^.

During the coronavirus disease (COVID-19) pandemic, governments introduced various policies to prevent and control the spread of the pandemic. World Health Organization (WHO) advocated that people wear masks and maintain a “one-meter line” social distance, and schools adopted online teaching, questioning, and tests instead of offline mode during the pandemic^22,23^. As of 2022, there have been three rounds of COVID-19 outbreaks in China, namely from January to March 2020, from March to May 2022, and at the end of 2022^24^. During the period from April 2020 to August 2021 after the first-round outbreak (hereinafter referred to as the early-COVID-19 period), Chinese colleges implemented many precautionary measures against the COVID-19 pandemic, such as setting barriers in the canteen, appealing to keeping social distance and conducting online teaching^25^. These measures reduced college students' social contact and may have an impact on their social phobia. Studies in developed countries showed that COVID-19 pandemic prevention and control measures may, on the one hand, alleviate the social phobia of college students in the short term by removing the social stimuli that originally triggered their fears (e.g., shared meals, and club activities); but on the other hand, they may be at risk of increased social phobia symptoms when they resume normal social interaction after the outbreak^26–28^. However, the impact of COVID-19 prevention and control measures on the social phobia of Chinese college students is still unclear.

This study aims to investigate the influencing factors of social phobia among Chinese college students and to tentatively explore the impact of COVID-19 prevention measures on social phobia in early-COVID-19 period.

## Methods

### Study design and participants

We conducted this cross-sectional survey from August 13 to 23, 2021, by inviting Chinese college students to participate in a web-based survey through the Wenjuanxing platform. We published recruitment notices on Chinese Internet social platforms (WeChat, QQ, and Baidu Post Bar). Risk control measures were taken during the data collection process, for example, the questionnaire was anonymous, all information was kept strictly confidential, and respondents had the right to refuse to answer. Each respondent who completed and submitted the questionnaire was paid 2 China Yuan as a subsidy. Inclusion criteria: Chinese college students (including junior college students, undergraduates, and postgraduates). Out of ethical considerations and control of potential confounding factors, we have developed exclusion criteria: (1) Under 18 or over 35 years old; (2) Pregnant or lactating women; (3) Suffering from serious physical or mental diseases and not yet recovered; (4) Taking psychotropic drugs in recent 2 weeks.

### Measurement of social phobia

We used the Peters short form of the Social Interaction Anxiety Scale and Social Phobia Scale (SIAS-6/SPS-6)^29^ to measure the social phobia score of college students in pre- and early-COVID-19 periods. Many studies from different populations and countries have demonstrated the satisfactory reliability and validity of this scale. The Cronbach's alpha coefficients of the SIAS-6 and SPS-6 in the Chinese university sample, were 0.742 and 0.868, respectively^30–32^. Each of the 12 items used a 5-point Likert scale response. A summative score was calculated from these 12 items (0–48 points), whereby a higher score reflects a higher degree of social phobia.

Respondents were asked to fill in SIAS-6/SPS-6 according to their status in early-COVID-19 period (August 2021, current date) and pre-COVID-19 period (before December 2019, retrospectively collected) respectively, representing their self-reported early- and pre-COVID-19 social phobia scores, the latter of which was scored retrospectively. Retrospective self-reported change in social phobia was assessed based on the difference between the early- and pre-COVID-19 social phobia scores. A reduction in social phobia score was defined as a difference less than 0, an unchanged score was defined as a difference equal to 0, and an increase was defined as a difference greater than 0.

### Questionnaire content

**Figure 1:**
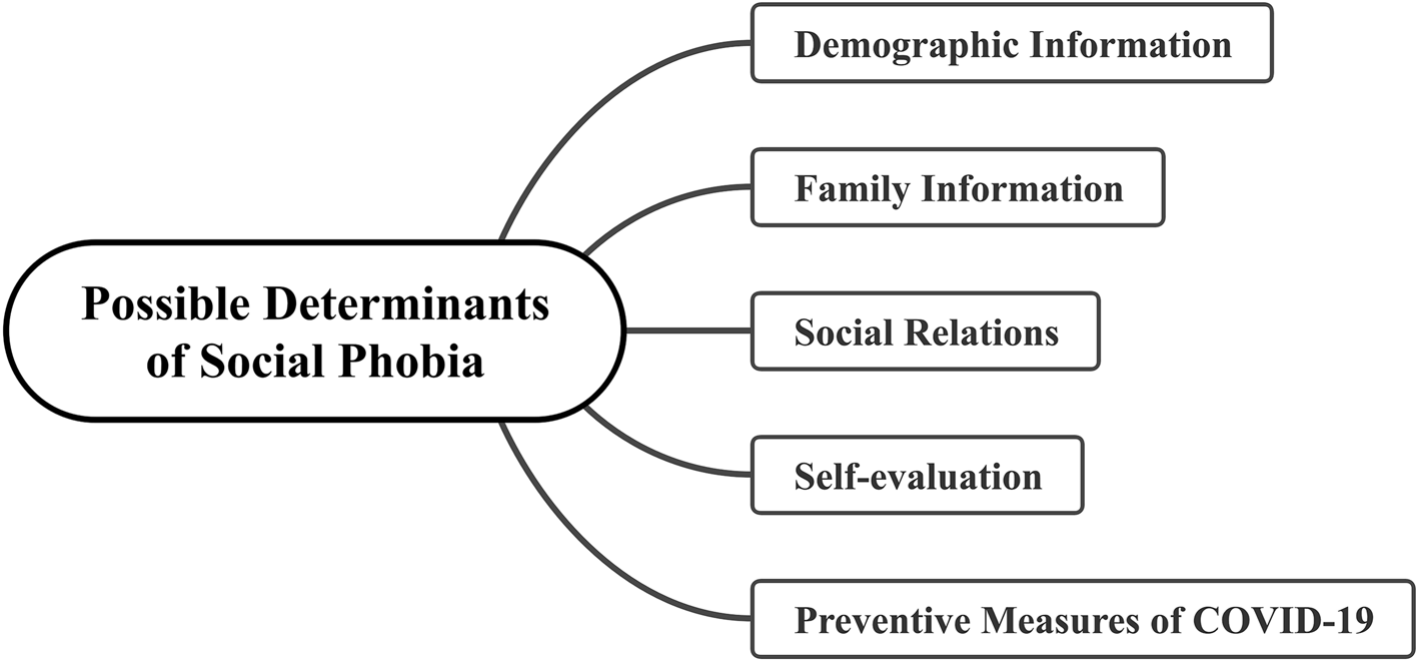
Conceptual framework of possible influencing factors of social phobia.

The questionnaire included two SIAS-6/SPS-6 scales (for pre- and early-COVID-19 periods) and five types of factors that may affect students’ social phobia status (Fig. 1), including demographic information, family information, social relations, self-evaluation, and subjective feelings about the impact of COVID-19’s preventive measures on social phobia.

Demographic information included gender, nationality, height, weight, registered residence, region (urban or rural), college type (according to China's “985 Project”^33^, universities were classified into 985 universities and non-985 universities, and the former was a group of Chinese high-level universities), grade, major, grade point average (GPA) ranking, and weekly exercise frequency (only consider a physical activity or exercise ≥ 1 h). Body mass index (BMI) was calculated and divided into underweight (BMI < 18.5), normal (18.5 ≤ BMI < 24.0), overweight (24.0 ≤ BMI < 28.0), and obesity (BMI ≥ 28.0) based on the Chinese population standard. According to the National Bureau of Statistics of China’s economic zone division^34^, a sum of 31 provinces, autonomous zones, and municipalities in China were divided into eastern, central, western, and northeast economic zones. The economic level of Macao and Taiwan was adjacent to the Eastern zone, thus incorporated into the Eastern zone for analysis. Family information included a family's monthly income per capita, family structure, parenting style, closeness of family's relationship, the number of siblings, and childhood adversity experience. Social relations included whether being in love, the number of love affairs, whether having been participated in student organizations, and childhood bullying experience. Self-evaluation included anxious feelings when one has yet to check the message or turn on the mobile phone for a period (reflecting the degree of mobile phone dependence), satisfaction of self-appearance, self-assessment of mental health, and degree of trust in strangers. Subjective feelings about the impact of COVID-19 preventive measures on social phobia included respondents' belief about wearing a mask, setting a baffle on the canteen dining table, keeping a one-meter social distance, vaccination against COVID-19, online presentation, or online asking or answering questions could alleviate or aggravate the social phobia degree.

According to data provided by the National Health Commission of the People's Republic of China and the National Bureau of Statistics, the cumulative prevalence of COVID-19 in Chinese mainland was only 6.68/100,000 (94,260/1,411,778,724) as of August 12th, 2021^35,36^, when almost no respondents were diagnosed with COVID-19. Therefore, we did not investigate whether the respondent was diagnosed with COVID-19.

### Quality control methods

Quality control procedures were carried out collaboratively by two data managers, and a data checker verified the correctness of the quality control process. Specifically, data quality checks restricted responses to one occurrence per social media platform, electronic device, and Internet Protocol (IP) address. Furthermore, respondents were excluded from the database if the questionnaires showed: (1) Logic errors, (2) identical answers, (3) completion time of less than 90 s, (4) height below 150 cm, and (5) weight under 30 kg. Respondents with a body mass index (BMI) of ≥ 35 kg/m^2^ were considered as an error, and this was modified by dividing weight by 2. This adjustment was made to account for the use of jin as a unit of measurement for weight in China (twice the value of kilograms). Although the questionnaire was labeled in kilograms, some respondents might still habitually provide their weight in jin.

### Ethics statement

All subjects gave their informed consent for inclusion before they participated in the study. The study was conducted in accordance with the Declaration of Helsinki, and the protocol was approved by the Peking University Medical Ethics Committee (IRB00001052-21054, June 10, 2021).

### Statistical analyses

Both the Kolmogorov–Smirnov test and Shapiro–Wilk test showed that college students’ SIAS-6/SPS-6 scores were not normally distributed (*P* < 0.001), so we used median (P_50_) and quartile (P_25_, P_75_) to describe their distribution. A multivariable binary logistic regression model was used to analyze the influencing factors of college students’ social phobia. Since there was no threshold for social phobia diagnosis in SIAS-6/SPS-6, we divided the current social phobia score into 2 groups of low score (0–10, n = 956) and high score (11–48, n = 903) according to the median as the outcome variable for best statistical power. Independent variables included demographic information, family information, social relations, and self-evaluation. We used the Akaike Information Criterion (AIC) to select variables by stepwise regression. McFadden’s R^2^ was used to evaluate the model's goodness of fit (generally > 0.2 could be considered to have a satisfied goodness of fit). Generalized variance inflation factors (GVIF) were used to evaluate the existence of multicollinearity (> 5 indicated multicollinearity). Subgroup analyses were conducted based on gender, region, and whether the respondent was the only child. Sensitivity analyses were conducted from two perspectives: variable selection methods and outcome variable classification criteria. The following 3 methods were used to select variables based on AIC: (1) backward regression; (2) forward regression; and (3) stepwise regression first, followed by the optimal subset regression. (Optimal subset regression was not used directly because it was limited by computational complexity and could only calculate all possible subsets of up to 15 variables. However, this study had up to 24 variables, therefore, the stepwise regression was preliminarily used to reduce the number of variables included, and then the optimal subset regression was used to obtain the optimal regression model.) In addition, the current social phobia scores were divided into ordinal variables according to tertiles (P_33_, P_67_) and quartiles (P_25_, P_50_, P_75_), respectively, as outcome indicators for ordinal logistic regression in sensitivity analyses to reflect the robustness of regression results.

A multivariable three-level ordinal logistic regression model was used to analyze the influencing factors of retrospective self-reported change in college students’ social phobia between early- and pre-COVID-19 periods. The outcome was an ordinal three-level variable: social phobia scale score increased (difference > 0), unchanged (difference = 0), and reduced (difference < 0), as defined previously. The subjective feelings about the impact of COVID-19’s preventive measures on social phobia were considered independent variables. Demographic information, family information, social relations, and self-evaluation were used as covariates. We also used AIC to select variables by stepwise regression.

Microsoft Excel was used to establish the database. R 4.1.2 (R Statistics, Vienna, Austria) was used for statistical analyses, and a two-tailed *P* value < 0.05 was viewed as statistically significant.

## Results

### Participant characteristics

A sum of 2469 questionnaires was collected, of which 1859 were valid, with an effective rate of 75.29%. In the study sample, just over half were males and nearly all the students were of Han ethnicity, about two-thirds were from urban areas. 49.0% were from the eastern zone, 25.9% were from the central zone, 20.5% were from the western zone, and 4.6% were from the northeast zone. The sample population composition ratio in this study was close to the college-age young adults in the 2020 Population Census of China in terms of gender, ethnicity, and economic zone^37^, therefore, it had a certain representativeness. Detailed characteristics of the respondents are shown in Table 1 below.

**Table 1:** Respondent characteristics (n = 1859). ^a^1 CNY = 0.1543 USD on August 14, 2021. *BMI* body mass index, *GPA* grade point average, *CNY* China Yuan, *USD* United States dollar.

Characteristic	Total n (%)	Social phobia score median (P_25_–P_75_)	Characteristic	Total n (%)	Social phobia score median (P_25_–P_75_)
Demographic information			Childhood bullying experience		
Gender			Never	756 (40.7)	5.0 (1.0–12.0)
Male	1002 (53.9)	12.0 (4.0–24.8)	Seldom	709 (38.1)	10 (4–20)
Female	857 (46.1)	8.0 (3.0–18.0)	Sometimes	326 (17.5)	23.5 (14.0–31.0)
Nation			Often	68 (3.7)	32.5 (21.0–38.5)
Ethnic Han	1763 (94.8)	10.0 (3.0–22.0)	Family Information		
Ethnic minorities	96 (5.2)	9.5 (2.8–16.0)	Monthly household income per capita^a^		
Economic zone			< 2000 CNY	108 (5.8)	12.0 (5.0–23.2)
Eastern	911 (49.0)	10.0 (3.0–22.0)	(< 309 USD)		
Central	481 (25.9)	11.0 (4.0–22.0)	2000–5000 CNY	527 (28.3)	12.0 (4.0–22.0)
Western	382 (20.5)	11.0 (4.0–21.0)	(309–772 USD)		
Northeast	85 (4.6)	6.0 (2.0–12.0)	5000–10,000 CNY	720 (38.7)	10.0 (3.0–21.0)
Region			(772–1543 USD)		
Urban	1257 (67.6)	10.0 (3.0–22.0)	> 10,000 CNY	504 (27.1)	7.50 (2.0–20.0)
Rural	602 (32.4)	11.0 (4.0–21.0)	(> 1543 USD)		
College type			Family structure		
Non-985	1307 (70.3)	11.0 (3.0–23.0)	Two-parents family	1698 (91.3)	9.0 (3.0–20.8)
985	552 (29.7)	9.0 (3.0–18.2)	Single parent family	104 (5.6)	19.0 (8.8–28.0)
Grade			Restructured family	37 (2.0)	23.0 (14.0–33.0)
1	229 (12.3)	12.0 (5.0–23.0)	Others	20 (1.1)	15.0 (8.0–28.2)
2	600 (32.3)	11.0 (3.0–24.0)	Parenting style		
3	688 (37.0)	10.0 (3.0–21.0)	Authoritative	1293 (69.6)	8.0 (3.0–20.0)
4 or 5	277 (14.9)	8.0 (3.0–19.0)	Authoritarian	233 (12.5)	16.0 (7.0–26.0)
Postgraduate	65 (3.5)	6.0 (2.0–12.0)	Neglectful	295 (15.9)	12.0 (5.0–23.5)
Major			Permissive	38 (2.0)	16.0 (7.2–28.8)
Literature or history	345 (18.6)	10.0 (3.0–21.0)	Closeness of family relationship		
Science or technology	911 (49.0)	11.0 (3.0–23.0)	Close	1405 (75.6)	8.0 (3.0–19.0)
Medicine	342 (18.4)	10.0 (4.0–20.0)	General	411 (22.1)	16.0 (8.0–27.0)
Economy or management	177 (9.5)	9.0 (3.0–17.0)	Alienated	43 (2.3)	20.0 (12.5–25.0)
Others	84 (4.5)	14.0 (3.0–22.2)	Number of siblings		
GPA ranking			0	873 (47.0)	8.0 (3.0–19.0)
Top 20%	323 (17.4)	8.0 (3.0–16.0)	1	676 (36.4)	11.0 (4.0–21.0)
20–40%	731 (39.3)	10.0 (3.0–21.0)	2	253 (13.6)	16.0 (7.0–28.0)
40–60%	573 (30.8)	11.0 (4.0–23.0)	≥ 3	57 (3.1)	11.0 (4.0–22.0)
60–80%	179 (9.6)	10.0 (3.0–23.5)	Childhood adversity experience		
After 80%	53 (2.9)	17.0 (8.0–25.0)	Never	756 (40.7)	5.0 (1.0–12.0)
BMI			Seldom	709 (38.1)	10 (4–20)
Normal	1198 (64.4)	10.0 (3.0–22.0)	Sometimes	326 (17.5)	23.5 (14.0–31.0)
Overweight	193 (10.4)	9.0 (3.0–20.0)	Often	68 (3.7)	32.5 (21.0–38.5)
Obesity	57 (3.1)	13.0 (6.0–28.0)	Self-evaluation		
Underweight	411 (22.1)	10.0 (4.0–21.0)	Mobile phone dependence		
Exercise frequency			No	462 (24.9)	4.0 (0.0–8.0)
< Once a week	438 (23.6)	14.0 (6.0–23.0)	General	863 (46.4)	10.0 (4.0–21.0)
1–2 times a week	882 (47.4)	12.0 (4.0–24.0)	Yes	534 (28.7)	18.0 (10.0–29.0)
≥ 3 times a week	539 (29.0)	6.0 (2.0–14.0)	Appearance satisfaction		
Social relations			Not very satisfied	317 (17.1)	17.0 (9.0–26.0)
Being in love now			Moderately satisfied	858 (46.2)	11.0 (5.0–21.0)
No	958 (51.5)	11.0 (4.0–22.0)	Quite satisfied	684 (36.8)	6.0 (2.0–17.0)
Yes	901 (48.5)	9.0 (3.0–21.0)	Mental health self-assessment		
Number of love affairs			Not very healthy	116 (6.2)	21.0 (12.0–30.0)
0	445 (23.9)	11.0 (4.0–21.0)	Moderately healthy	626 (33.7)	17.0 (9.0–26.0)
1–2	1233 (66.3)	10.0 (3.0–22.0)	Quite healthy	1117 (60.1)	6.0 (2.0–15.0)
≥ 3	181 (9.7)	9.0 (3.0–18.0)	Degree of trust in strangers		
Student organizations participation			Not very trust	585 (31.5)	12.0 (5.0–21.0)
Ever	1373 (73.9)	9.0 (3.0–21.0)	Moderately trust	845 (45.5)	9.0 (3.0–21.0)
Never	486 (26.1)	13.0 (5.0–24.0)	Quite trust	429 (23.1)	8.0 (3.0–25.0)

### Influencing factors of college students’ social phobia

The GVIF of all variables in the main analysis, subgroup analyses, and sensitivity analyses were less than 1.66, indicating the absence of multicollinearity. McFadden’s R^2^ of all models in the main analysis and subgroup analyses were greater than 0.27, reflecting a satisfied goodness of fit.

Multivariable logistic regression results showed that risk factors for social phobia were: lower GPA (OR: 1.40–2.36, as compared with those in the top quintile), higher frequencies of bullying experience (OR: 1.31–3.44, vs non-bullied students), permissive parents (OR = 2.82, vs authoritative parents), general family relationships (OR = 1.35, vs close family relationships), having 1 or 2 siblings (OR: 1.35–2.18, vs no sibling), higher frequencies of childhood adversity (OR: 1.63–11.35, vs never experienced childhood adversity), and mobile phone dependence (OR: 2.32–5.12).

Protective factors for social phobia were female gender (OR = 0.63), being a postgraduate or grade 4/5 student (OR: 0.38–0.60, vs grade 1), being quite satisfied with their appearance (OR = 0.64, vs not very satisfied), self-perceived mentally quite healthy (OR = 0.37, vs not very healthy), and moderately trust in strangers (OR = 0.72, vs not very trust) (Table 2).

**Table 2:** Logistic regression of influencing factors for social phobia (main analysis). The outcome variable was divided by median into 2 groups of low score (0–10, n = 956) and high score (11–48, n = 903). McFadden’s R^2^ = 0.285. GVIF for all variables is less than 1.58. *OR* odds ratio, *CI* confidence interval, *GPA* grade point average.

Factor	OR (95% CI)	*P* value
Demographic information		
Gender (female vs male)	0.63 (0.50–0.79)	< 0.001
Region (rural vs urban)	0.82 (0.63–1.07)	0.136
Grade (vs 1)		
2	0.82 (0.56–1.21)	0.322
3	0.78 (0.54–1.14)	0.206
4 or 5	0.60 (0.39–0.94)	0.025
Postgraduate	0.38 (0.19–0.78)	0.008
GPA ranking (vs top 20%)		
20–40%	1.40 (1.00–1.96)	0.049
40–60%	1.47 (1.04–2.09)	0.030
60–80%	1.04 (0.65–1.68)	0.867
After 80%	2.36 (1.07–5.21)	0.033
Exercise frequency (vs < once a week)		
1–2 times a week	1.14 (0.85–1.52)	0.397
≥ 3 times a week	0.82 (0.59–1.15)	0.246
Social relations		
Never participate in student organizations (vs ever)	1.26 (0.97–1.66)	0.089
Childhood bullying experience (vs never)		
Seldom	1.31 (1.00–1.71)	0.047
Sometimes	2.91 (1.95–4.33)	< 0.001
Often	3.44 (1.36–8.65)	0.009
Family Information		
Parenting style (vs authoritative)		
Authoritarian	1.08 (0.75–1.56)	0.691
Neglectful	0.90 (0.65–1.24)	0.511
Permissive	2.82 (1.21–6.60)	0.017
Closeness of family relationship (vs close)		
General	1.35 (1.01–1.82)	0.044
Alienated	1.26 (0.49–3.21)	0.627
Number of siblings (vs 0)		
1	1.35 (1.04–1.75)	0.027
2	2.18 (1.52–3.14)	< 0.001
≥ 3	1.08 (0.55–2.14)	0.814
Childhood adversity experience (vs never)		
Seldom	1.63 (1.23–2.14)	< 0.001
Sometimes	2.64 (1.84–3.80)	< 0.001
Often	11.35 (4.47–28.79)	< 0.001
Self-evaluation		
Mobile phone dependence (vs no)		
General	2.32 (1.72–3.13)	< 0.001
Yes	5.12 (3.63–7.22)	< 0.001
Appearance satisfaction (vs not very satisfied)		
Moderately satisfied	0.83 (0.59–1.17)	0.293
Quite satisfied	0.64 (0.43–0.93)	0.021
Mental health self-assessment (vs not very healthy)		
Moderately healthy	0.76 (0.43–1.35)	0.348
Quite healthy	0.37 (0.21–0.66)	< 0.001
Degree of trust in strangers (vs not very trust)		
Moderately trust	0.72 (0.55–0.94)	0.016
Quite trust	0.99 (0.71–1.36)	0.929

### Subgroup analyses by gender, region, and whether the respondent was the only child

The influencing factors of Chinese students’ social phobia were diverse by gender, region, and whether he/she was the only child.

For male students, risk factors for social phobia were sometimes or often being bullied (OR: 3.52–4.56, vs never been bullied), middle-high household income (OR = 2.48, vs low household income), general family relationships (OR = 1.68, vs close family relationships), having 1 or 2 siblings (OR: 1.43–2.55, vs no sibling), higher frequencies of childhood adversity (OR: 1.59–8.37, vs never experienced childhood adversity), and mobile phone dependence (OR: 2.27–4.10); protective factors were being from northeast of China (OR = 0.27, vs eastern China), being a postgraduate student (OR = 0.22, vs grade 1), and being quite satisfied with their appearance (OR = 0.50, vs not very satisfied).

For female students, risk factors for social phobia were sometimes being bullied (OR = 2.44, vs never been bullied), having 2 siblings (OR = 1.84, vs no sibling), higher frequencies of childhood adversity (OR: 1.78–21.46, vs never experienced childhood adversity), and mobile phone dependence (OR: 2.61–7.56); protective factor was self-perceived mentally quite healthy (OR = 0.17, vs not very healthy) (Table 3).

**Table 3:** Logistic regression of the influencing factors of social phobia between males and females (subgroup analysis). ^a^McFadden's R^2^ = 0.308. GVIF for all variables is less than 1.66. ^b^McFadden's R^2^ = 0.271. GVIF for all variables is less than 1.51. – for variables without statistical significance that were removed by stepwise regression. *OR* odds ratio, *CI* confidence interval.

Factor	Male (n = 1002)^a^		Female (n = 857)^b^	
	OR (95% CI)	*P* value	OR (95% CI)	*P* value
Demographic information				
Economic zone (vs Eastern)				
Central	0.78 (0.53–1.14)	0.193	–	–
Western	0.96 (0.62–1.48)	0.862	–	–
Northeast	0.27 (0.09–0.77)	0.014	–	–
Grade (vs 1)				
2	0.83 (0.49–1.38)	0.465	–	–
3	0.99 (0.59–1.66)	0.983	–	–
4 or 5	0.59 (0.32–1.10)	0.096	–	–
Postgraduate	0.22 (0.08–0.64)	0.006	–	–
Exercise frequency (vs < once a week)				
1–2 times a week	1.41 (0.91–2.19)	0.121	–	–
≥ 3 times a week	0.72 (0.44–1.17)	0.183	–	–
Social relations				
Being in love now (yes vs no)	0.76 (0.55–1.06)	0.108	–	–
Never participate in student organizations (vs ever)	1.40 (0.96–2.06)	0.081	1.39 (0.95–2.04)	0.091
Childhood bullying experience (vs never)				
Seldom	1.10 (0.76–1.60)	0.604	1.46 (1.00–2.15)	0.052
Sometimes	3.52 (2.00–6.22)	< 0.001	2.44 (1.37–4.34)	0.002
Often	4.56 (1.20–17.35)	0.026	3.09 (0.76–12.53)	0.113
Family information				
Monthly household income per capita (vs < 309 USD)				
309–772 USD	1.99 (0.95–4.19)	0.070	–	–
772–1543 USD	2.48 (1.17–5.22)	0.017	–	–
> 1543 USD	1.72 (0.80–3.67)	0.164	–	–
Closeness of family relationship (vs close)				
General	1.68 (1.10–2.56)	0.016	–	–
Alienated	1.92 (0.47–7.85)	0.363	–	–
Number of siblings (vs 0)				
1	1.43 (1.01–2.03)	0.045	1.13 (0.78–1.65)	0.521
2	2.55 (1.49–4.35)	< 0.001	1.84 (1.14–2.98)	0.013
≥ 3	1.27 (0.44–3.69)	0.664	0.70 (0.28–1.76)	0.454
Childhood adversity experience (vs never)				
Seldom	1.59 (1.07–2.35)	0.021	1.78 (1.20–2.64)	0.004
Sometimes	2.84 (1.73–4.67)	< 0.001	2.40 (1.41–4.10)	0.001
Often	8.37 (2.77–25.28)	< 0.001	24.16 (2.99–195.48)	0.003
Self-evaluation				
Mobile phone dependence (vs no)				
General	2.27 (1.53–3.38)	< 0.001	2.61 (1.62–4.20)	< 0.001
Yes	4.10 (2.54–6.63)	< 0.001	7.56 (4.54–12.61)	< 0.001
Appearance satisfaction (vs not very satisfied)				
Moderately satisfied	0.72 (0.42–1.22)	0.219	–	–
Quite satisfied	0.50 (0.28–0.89)	0.019	–	–
Mental health self-assessment (vs not very healthy)				
Moderately healthy	1.17 (0.53–2.62)	0.693	0.44 (0.19–1.03)	0.059
Quite healthy	0.64 (0.29–1.43)	0.273	0.17 (0.08–0.39)	< 0.001
Degree of trust in strangers (vs not very trust)				
Moderately trust	–	–	0.70 (0.48–1.03)	0.072
Quite trust	–	–	1.12 (0.71–1.76)	0.637

For students from urban areas, risk factors for social phobia were never participating in student organizations (OR = 1.66), sometimes or often being bullied (OR: 2.07–3.37, vs never been bullied), low-middle household income (OR = 2.79, vs low household income), permissive parents (OR = 4.67, vs authoritative parents), having 1 or 2 siblings (OR: 1.41–2.82, vs no sibling), higher frequencies of childhood adversity (OR: 1.73–12.23, vs never experienced childhood adversity), and mobile phone dependence (OR: 2.04–4.64); protective factors were female gender (OR = 0.73), being from northeast of China (OR = 0.40, vs eastern China), and self-perceived mentally quite healthy (OR = 0.30, vs not very healthy).

For students from rural areas, risk factors for social phobia were sometimes been bullied (OR = 5.93, vs never been bullied), often experienced childhood adversity (OR = 19.78, vs never experienced childhood adversity), and mobile phone dependence (OR: 3.05–5.89); protective factors were female gender (OR = 0.58), being a postgraduate or grade 4/5 student (OR: 0.22–0.38, vs grade 1), exercise ≥ 3 times a week (OR = 0.54, vs less than once a week), being quite satisfied with their appearance (OR = 0.48, vs not very satisfied), and moderately trust in strangers (OR = 0.59, vs not very trust) (Table 4).

**Table 4:** Logistic regression of the influencing factors of social phobia between urban and rural college students (subgroup analysis). ^a^McFadden's R^2^ = 0.294, GVIF for all variables is less than 1.58. ^b^McFadden's R^2^ = 0.287, GVIF for all variables is less than 1.53. – for variables without statistical significance that were removed by stepwise regression. *OR* odds ratio, *USD* United States dollar.

Factor	Urban (n = 1257)^a^		Rural (n = 602)^b^	
	OR (95% CI)	*P* value	OR (95% CI)	*P* value
Demographic information				
Gender (female vs male)	0.73 (0.55–0.96)	0.027	0.58 (0.38–0.89)	0.013
Economic zone (vs Eastern)				
Central	1.09 (0.77–1.56)	0.630	–	–
Western	0.95 (0.65–1.38)	0.792	–	–
Northeast	0.40 (0.20–0.79)	0.009	–	–
Grade (vs 1)				
2	–	–	0.66 (0.37–1.16)	0.149
3	–	–	0.65 (0.37–1.14)	0.136
4 or 5	–	–	0.38 (0.18–0.80)	0.011
Postgraduate	–	–	0.22 (0.06–0.75)	0.015
Exercise frequency (vs < once a week)				
1–2 times a week	–	–	1.12 (0.70–1.80)	0.642
≥ 3 times a week	–	–	0.54 (0.31–0.95)	0.034
Social relations				
Never participate in student organizations (vs ever)	1.66 (1.17–2.36)	0.004		
Childhood bullying experience (vs never)				
Seldom	1.15 (0.83–1.59)	0.402	1.47 (0.93–2.34)	0.102
Sometimes	2.07 (1.28–3.36)	0.003	5.93 (2.87–12.24)	< 0.001
Often	3.37 (1.13–10.04)	0.029	3.63 (0.64–20.48)	0.144
Family information				
Monthly household income per capita (vs < 309 USD)				
309–772 USD	2.79 (1.21–6.41)	0.016	–	–
772–1543 USD	2.11 (0.94–4.77)	0.072	–	–
> 1543 USD	1.69 (0.74–3.87)	0.212	–	–
Parenting style (vs authoritative)				
Authoritarian	1.38 (0.88–2.15)	0.156	–	–
Neglectful	1.01 (0.67–1.53)	0.965	–	–
Permissive	4.67 (1.44–15.17)	0.010	–	–
Number of siblings (vs 0)				
1	1.41 (1.03–1.93)	0.031	–	–
2	2.82 (1.75–4.55)	< 0.001	–	–
≥ 3	0.95 (0.26–3.40)	0.933	–	–
Childhood adversity experience (vs never)				
Seldom	1.73 (1.24–2.43)	0.001	1.52 (0.94–2.47)	0.090
Sometimes	3.60 (2.28–5.69)	< 0.001	1.50 (0.82–2.74)	0.183
Often	12.23 (4.38–34.17)	< 0.001	19.78 (2.17–180.54)	0.008
Self-evaluation				
Mobile phone dependence (vs no)				
General	2.04 (1.42–2.93)	< 0.001	3.05 (1.76–5.27)	< 0.001
Yes	4.64 (3.07–7.02)	< 0.001	5.89 (3.12–11.13)	< 0.001
Appearance satisfaction (vs not very satisfied)				
Moderately satisfied	1.10 (0.71–1.70)	0.670	0.60 (0.34–1.08)	0.089
Quite satisfied	0.79 (0.50–1.26)	0.331	0.48 (0.24–0.94)	0.032
Mental health self-assessment (vs not very healthy)				
Moderately healthy	0.83 (0.41–1.65)	0.586	0.85 (0.32–2.27)	0.746
Quite healthy	0.30 (0.15–0.61)	< 0.001	0.51 (0.19–1.38)	0.186
Degree of trust in strangers (vs not very trust)				
Moderately trust	–	–	0.59 (0.37–0.93)	0.024
Quite trust	–	–	0.59 (0.32–1.08)	0.085

For students who were the only child in the family, risk factors for social phobia were sometimes been bullied (OR = 2.32, vs never been bullied), higher frequencies of childhood adversity (OR: 1.52–12.23, vs never experienced childhood adversity), high level of mobile phone dependence (OR: 5.19); protective factors were being from northeast of China (OR = 0.34, vs eastern China), and being quite satisfied with their appearance (OR = 0.34, vs not very satisfied).

For students with siblings, risk factors for social phobia were middle-level GPA (OR: 1.87–2.03, as compared with those in the top quintile), underweight (OR = 1.66), sometimes or often being bullied (OR: 3.63–4.57, vs never been bullied), general family relationships (OR = 1.73, vs close family relationships), having 2 siblings (OR: 1.72, vs having 1 sibling), seldom or often experienced childhood adversity (OR: 1.67–11.63, vs never experienced childhood adversity), and mobile phone dependence (OR: 2.97–5.01); protective factors were female gender (OR = 0.42), being from rural areas (OR = 0.70), overweight (OR = 0.53), being quite satisfied with their appearance (OR = 0.57, vs not very satisfied), and self-perceived mentally quite healthy (OR = 0.26, vs not very healthy) (Table 5).

**Table 5:** Logistic regression of the influencing factors of social phobia between the only child and child with siblings (subgroup analysis). ^a^McFadden's R^2^ = 0.312. GVIF for all variables is less than 1.57. ^b^McFadden's R^2^ = 0.285. GVIF for all variables is less than 1.61. – for variables without statistical significance that were removed by stepwise regression. / for not applicable. *OR* odds ratio, *CI* confidence interval, *BMI* body mass index, *GPA* grade point average, *USD* United States dollar.

Factor	The only child (n = 873)^a^		Child with siblings (n = 986)^b^	
	OR (95% CI)	*P* value	OR (95% CI)	*P* value
Demographic information				
Gender (female vs male)	–	–	0.42 (0.30–0.59)	< 0.001
Nation (ethnic minorities vs ethnic Han)	2.05 (0.95–4.46)	0.069	–	–
Economic zone (vs Eastern)				
Central	0.80 (0.51–1.26)	0.338	–	–
Western	0.77 (0.48–1.24)	0.284	–	–
Northeast	0.34 (0.17–0.69)	0.003	–	–
Region (rural vs urban)	–	–	0.70 (0.51–0.98)	0.037
College type (985 vs non-985)	–	–	0.76 (0.53–1.10)	0.144
GPA ranking (vs top 20%)				
20–40%	–	–	2.03 (1.27–3.24)	0.003
40–60%	–	–	1.87 (1.14–3.08)	0.013
60–80%	–	–	1.47 (0.74–2.91)	0.275
After 80%	–	–	2.39 (0.91–6.32)	0.078
BMI (vs normal)				
Overweight	–	–	0.53 (0.30–0.93)	0.028
Obesity	–	–	1.68 (0.62–4.58)	0.308
Underweight	–	–	1.66 (1.13–2.44)	0.010
Exercise frequency (vs < once a week)				
1–2 times a week	0.99 (0.62–1.57)	0.954	–	–
≥ 3 times a week	0.66 (0.40–1.11)	0.120	–	–
Social relations				
Being in love now (yes vs no)	–	–	0.78 (0.57–1.08)	0.141
Never participate in student organizations (vs ever)	–	–	1.32 (0.92–1.89)	0.127
Childhood bullying experience (vs never)				
Seldom	1.33 (0.89–1.99)	0.165	1.13 (0.79–1.63)	0.504
Sometimes	2.32 (1.29–4.18)	0.005	3.63 (2.06–6.40)	< 0.001
Often	2.43 (0.70–8.40)	0.161	4.57 (1.11–18.74)	0.035
Family information				
Monthly household income per capita (vs < 309 USD)				
309–772 USD	2.22 (0.85–5.82)	0.104	–	–
772–1543 USD	1.61 (0.63–4.15)	0.322	–	–
> 1543 USD	1.19 (0.46–3.11)	0.716	–	–
Closeness of family relationship (vs close)				
General	–	–	1.73 (1.17–2.54)	0.006
Alienated	–	–	1.12 (0.39–3.23)	0.830
Number of siblings (vs 1)				
2	/	/	1.72 (1.19–2.48)	0.004
≥ 3	/	/	0.85 (0.43–1.70)	0.654
Childhood adversity experience (vs never)				
Seldom	1.52 (1.00–2.31)	0.049	1.67 (1.15–2.45)	0.008
Sometimes	5.09 (2.95–8.78)	< 0.001	1.46 (0.89–2.39)	0.137
Often	12.23 (3.71–40.32)	< 0.001	11.63 (2.51–53.90)	0.002
Self-evaluation				
Mobile phone dependence (vs no)				
General	1.49 (0.95–2.35)	0.086	2.97 (1.96–4.50)	< 0.001
Yes	5.19 (3.15–8.54)	< 0.001	5.01 (3.09–8.14)	< 0.001
Appearance satisfaction (vs not very satisfied)				
Moderately satisfied	1.02 (0.48–2.15)	0.962	0.86 (0.54–1.36)	0.523
Quite satisfied	0.34 (0.16–0.71)	0.004	0.57 (0.34–0.96)	0.035
Mental health self-assessment (vs not very healthy)				
Moderately healthy	–	–	0.54 (0.22–1.34)	0.182
Quite healthy	–	–	0.26 (0.11–0.66)	0.004
Degree of trust in strangers (vs not very trust)				
Moderately trust	–	–	0.76 (0.53–1.08)	0.125
Quite trust	–	–	1.18 (0.75–1.87)	0.475

### Sensitivity analyses

The three different variable selection methods, namely the backward regression, forward regression, and stepwise regression followed by optimal subset regression, all provided models that were completely consistent with the main analysis (stepwise regression), indicating that the variable selection method in this study did not affect the robustness of the regression results. The results based on two different outcome variable classification criteria, namely the tertiles and quartiles (Supplementary Tables S1, S2), are basically consistent with the main analysis, indicating that the regression results were robust for different outcome variable classification criteria. Very few variables with significant margins transition to insignificant in sensitivity analysis results, which may be due to the loss of statistical power in the division of ordered multinomial outcomes compared to binary classification outcomes in the regression.

### College students’ social phobia score in pre- and early-COVID-19 periods

Before the outbreak of COVID-19, the college students’ average social phobia score was 12.3 ± 11.9, with a median (P_25_–P_75_) of 9 (2–20). During the COVID-19 pandemic, the average score was 13.4 ± 11.9, with a median (P_25_–P_75_) of 10 (3–21), as shown in Supplementary Fig. S1. During the pandemic, college students’ social phobia scores increased by 1.0 ± 6.4, with a median (P_25_–P_75_) of 0 (− 1 to 2), as shown in Supplementary Fig. S2. The paired Wilcoxon rank-sum test showed as statistically significant (*P* < 0.001). During the pandemic, 28.8% of respondents’ social phobia was alleviated (score reduced), 27.5% had no significant change (score unchanged), and 43.7% were intensified (score increased).

### Influencing factors of retrospective self-reported change between college students’ social phobia in pre- and early-COVID-19 periods

The proportions of respondents who considered wearing a mask, online asking or answering questions, online presentation, setting a baffle on the canteen dining table, keeping a one-meter social distance, and getting COVID-19 vaccinated alleviated their social phobia were 46.4%, 43.9%, 41.3%, 41.2%, 40.0%, and 33.1%, respectively. Nearly half of the respondents considered these measures to have no impact on their social phobia status. Only about 10% of the respondents considered these measures aggravated their social phobia (Supplementary Table S3).

Multivariable 3-level ordinal logistic regression showed that the potential risk factors for social phobia aggravation might be: thinking the table baffle did not affect social phobia (OR = 1.30, vs thinking that could alleviate social phobia), having 2 siblings (OR = 1.38, vs no sibling), and obesity (OR = 1.78). The potential protective factors might be thinking COVID-19 vaccination did not affect social phobia (OR = 0.73, vs thinking that could alleviate social phobia), exercise 1–2 times a week (OR = 0.80, vs less than once a week) (Supplementary Table S4).

## Discussion

This study is one of the few nationwide studies in China focusing on college students’ SP and its influencing factors during the COVID-19 pandemic. Infectious disease pandemics have a lasting impact on mental health^38,39^. The following study showed that psychological distress slowly declined over 28 months during an avian influenza pandemic^38^; another study showed that among those infected with SARS, the psychiatric effects were long-term^39^. Similarly, the COVID-19 pandemic led to dramatic changes in lifestyles around the world and has had detrimental effects on mental health^40^, and it appeared unlikely that mental health would return to pre-pandemic levels in the near future^41^. Our study showed that the social phobia level among Chinese college students slightly increased during the COVID-19 pandemic. This may be related to the increase in measures such as quarantine and lockdowns, avoidance of non-essential social interactions, and online learning during COVID-19, which reinforced social avoidance and took away the sense of well-being gained from interacting with others^42^.

Our study indicated that SP among Chinese college students was widely influenced by factors from individuals, families, social relations, and self-evaluations, and were diverse by gender, region, and whether he/she was the only child.

On the aspect of individuals, we found that senior students are associated with a decrease in the risk of SP. Concerning relevant studies in other countries, we found similar patterns to our research^43–45^. The possible reasons for this are that lower-grade students may live away from their parents for the first time, out of a comfortable social environment, and on the other hand, as they age and progress in learning, they might gradually adapt to new life and social environments^46^. The subgroup analysis showed that this protective effect mainly appears in rural students. This may be because the new students from rural areas have relatively little experience in urban university life, and with the growth of grades, life experience, and interpersonal relationships increase, alleviating their SP.

With regard to family and social relations, college students with permissive parents demonstrated a higher risk of SP than those from authoritative parents. It is possible that children with highly permissive parents may develop a self-centered personality that may contribute to poor interpersonal interactions out of the home, leading to a higher risk of SP^47^. But it is also possible that growing up with highly permissive parents will lead to the less independence in the child and lowered self-confidence in their social interactions^48^. We also found that the SP risk of the only child is lower than that of students with siblings, which was consistent with a study in China^47^. According to a study conducted in Canada, non-only children are more likely to have conflicts and social contagion (i.e. adolescents are more prone to mimic their siblings’ expressions) with their siblings, which has been associated with the onset and exacerbation of both anxiety and social phobia^49^. In subgroup analysis, the association between having siblings and SP was mainly manifested in urban students. This may be because urban families are relatively more independent from neighbors, so children may have more communication with siblings, but less with other peers, leading to SP. Childhood adversity and peer bullying experiences were risk factors for SP, which was consistent with studies in China and other countries^50,51^. Childhood adversity and peer bullying experience will lead to lower self-esteem and self-evaluation in adulthood and will produce distrust and insecurity towards others^51,52^, resulting in social phobia in interpersonal communication.

On the aspect of self-appraisal and self-image, our study showed that individuals who consider themselves mentally healthy had a lower risk of developing SP, and notably, this phenomenon was particularly pronounced among females, aligning with previous research findings^53^. We also found that the satisfaction of self-appearance was negatively associated with SP, suggesting that self-abasement due to their outer appearance may prefer avoiding social contact and developing SP^54^. Similar findings from a study in the United Kingdom also imply that negative self-imagery is associated with a higher degree of social disengagement and an increased risk of SP^55^. Besides, in our study, students with low level of interpersonal trust score higher on SP. A previous study showed that during a pandemic, the level of trust between people may be reduced, leading to negative psychological states such as interpersonal trust crisis, social phobia, and loneliness^56^, which might explain the aggravation of social phobia during the pandemic among college students in our study. Mobile phone dependence had a very high risk on SP, and this effect seemed to be universal since it existed in all subgroups of this study. An Indian undergraduates-based study also showed that students with social phobia were more unable to cut down on smartphone usage^57^. Many other studies also indicated that smartphones could both alleviate loneliness and increase anxiety when not available^58,59^.

Our descriptive study showed that more people believed that the COVID-19 prevention measures would alleviate SP, however, SP score had slightly increased during the pandemic. This might be due to the influence of unconsidered confounding factors. For example, the outbreak itself can increase anxiety levels in the population, which may aggravate SP. According to our regression results, setting canteen baffles was a potential protective factor against social phobia aggravation. A prior study indicated that individuals with SP were more likely to use voice or text media rather than visual media^60^, and the baffles blocked the visual media, which might alleviate their SP degree. Unfortunately, McFadden’s R^2^ of this regression model was only 0.011, suggesting that it had insufficient explanatory power and there were unknown influencing factors, which need further research in the future.

College administrators should pay more attention to the SP among students and can carry out screening, assessment, tracking, and early intervention of high-risk groups. Especially for freshmen (particularly boys from rural areas), students with lower GPA rankings, and students with other psychological disorders. In addition, eliminating campus bullying and promoting the reduction of smartphone dependence are also measures recommended for college administrators to prevent students from SP.

### Limitations

Recall bias in the retrospective collection of social phobia status in pre-COVID-19 period was the major limitation in our study. We were very cautious in interpreting the exploratory results of the retrospective self-reported change in social phobia between pre- and early-COVID-19 periods and have mainly focused our research on the influencing factors of the current social phobia status among college students. In addition, although SIAS-6/SPS-6 is simple and applicable to Chinese college students, it lacks a threshold for the diagnosis of social phobia, so we cannot calculate the prevalence of social phobia to compare with previous studies.

## Conclusions

Social phobia among Chinese college students was widely influenced by individuals, families, social relations, and self-evaluations, and was diverse by gender, region, and whether he/she was the only child. The results have a reference value for early screening and risk factor intervention of Chinese college students’ social phobia. Most participants subjectively believed that COVID-19 prevention and control measures could alleviate social phobia to a certain extent in early-COVID-19 period, but its real effect is still uncertain, and more in-depth research needs to be carried out in the future.

### Supplementary Information

Supplementary Information.

## Data Availability

The datasets generated during and/or analyzed during the current study are available from the corresponding author on reasonable request.

## Abbreviations

SP: Social phobia
COVID-19: Coronavirus disease
SIAS-6/SPS-6: The Peters short form of the Social Interaction Anxiety Scale and Social Phobia Scale
SIAS: Social Interaction Anxiety Scale
SPS: Social Phobia Scale
BMI: Body mass index
GPA: Grade point average
CNY: China Yuan
USD: United States dollar
OR: Odds ratio
CI: Confidence interval
IP: Internet Protocol
AIC: Akaike Information Criterion
GVIF: Generalized variance inflation factors
