# Supplementary material for: Factors affecting social phobia among Chinese college students in the context of COVID-19 pandemic: a cross-sectional study
Source: Sci Rep. 2023 Nov 28;13:20900. doi: 10.1038/s41598-023-48225-y (PMC10684864; doi:10.1038/s41598-023-48225-y)
Supplement: Supplementary file 1 — Supplementary Information. [file 41598_2023_48225_MOESM1_ESM.docx]

**Factors affecting social phobia among Chinese college students in the context of COVID-19 pandemic: a cross-sectional study**

Hai Lin^1,2,†^ , Ziming Yang^2,3,†^, Shanshan Huo^4^, Caixia Su^2^, Zhongsong Zhang^5^, Yingting Rao^1,2^ and Hui Yin^5,6,*^

^1^ Institute of Reproductive and Child Health, Peking University/ Key Laboratory of Reproductive Health, National Health Commission of the People's Republic of China, Beijing 100191, China

^2^ Department of Epidemiology and Biostatistics, School of Public Health, Peking University, Beijing 100191, China

^3^ Key Laboratory of Epidemiology of Major Diseases (Peking University), Ministry of Education, Beijing 100191, China.

^4^ Department of Health Policy and Management, School of Public Health, Peking University, Beijing 100191, China

^5^ School of Public Health, Peking University, Beijing 100191, China.

^6^ Institute of Global Health, Peking University, Beijing 100191, China.

^*^email: yinhui@pku.edu.cn

†Hai Lin and Ziming Yang have contributed equally to this work and should be considered co-first authors

**Supplementary material**

**Content**

**Supplementary Figure S1.** College students’ social phobia score in pre- and early-COVID-19 period.

**Supplementary Figure S2.** Retrospective self-reported change of college students’ social phobia score.

**Supplementary Table S1.** Ordinal logistic regression for the outcome variable divided by tertiles (sensitivity analysis).

**Supplementary Table S2.** Ordinal logistic regression for the outcome variable divided by quartiles (sensitivity analysis).

**Supplementary Table S3.** Subjective feelings of COVID-19’s preventive measures on social phobia (n=1859).

**Supplementary Table S4**. Ordinal logistic regression of influencing factors of retrospective self-reported change in college students’ social phobia in pre- and early-COVID-19 periods.


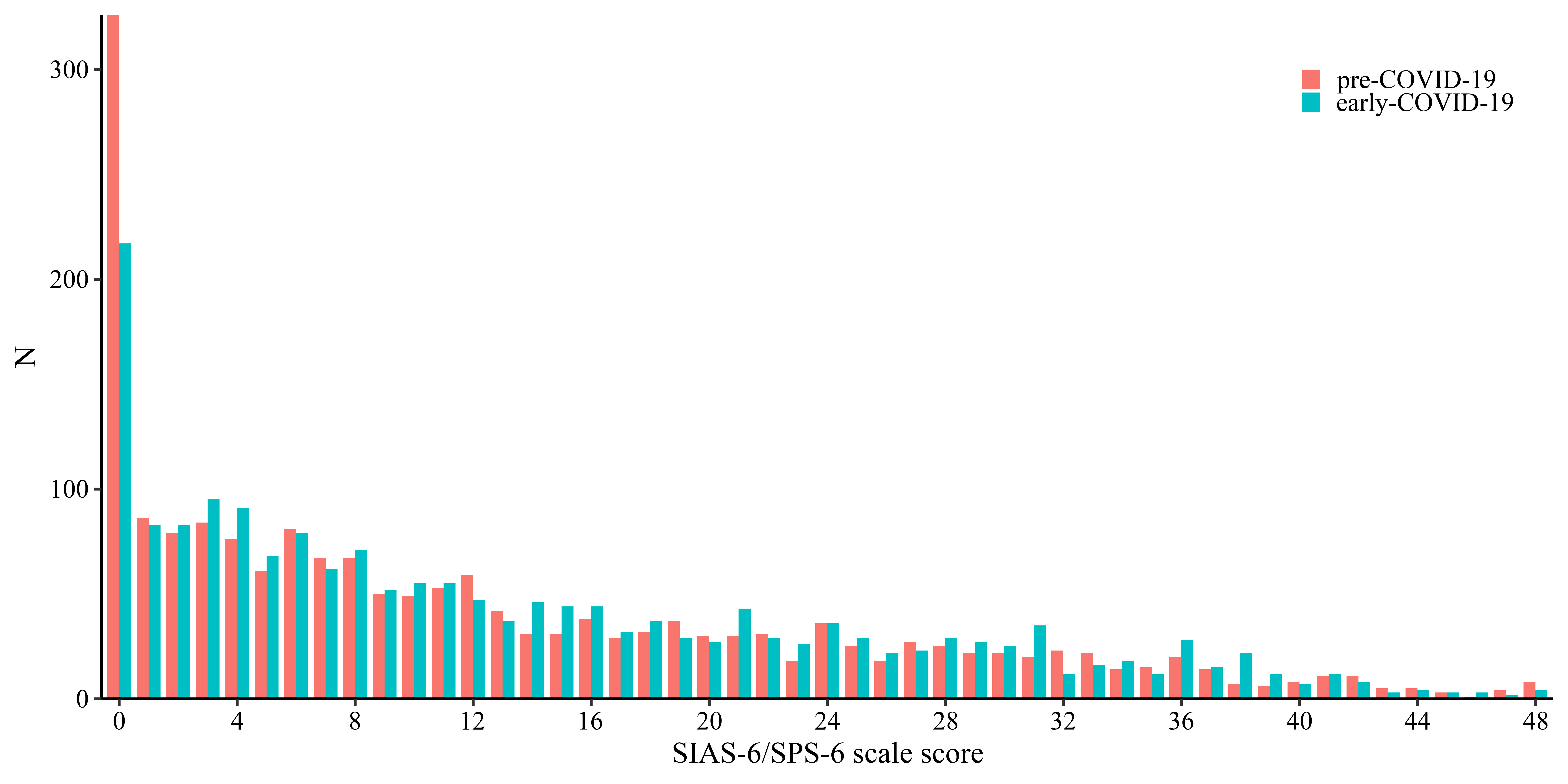


**Supplementary Figure S1**. College students’ social phobia score in pre- and early-COVID-19 periods.


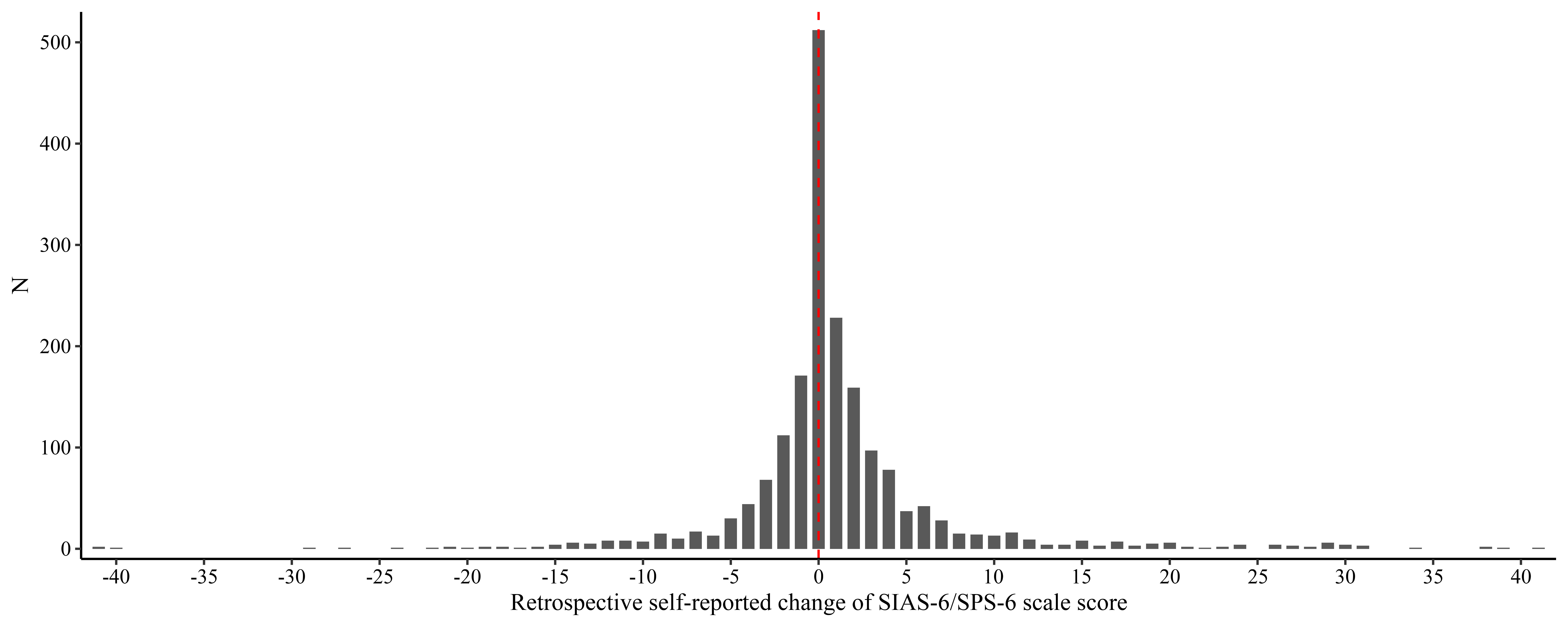


**Supplementary Figure S2**. Retrospective self-reported change of college students’ social phobia score.

| **Factor** | **OR (95%CI)** | ***P* Value** |
| --- | --- | --- |
| **Demographic information** |  |  |
| **Gender (female vs male)** | 0.68 (0.56~0.82) | <0.001 |
| **Economic zone (vs Eastern)** |  |  |
| Central | 0.95 (0.75~1.19) | 0.638 |
| Western | 0.77 (0.60~0.98) | 0.036 |
| Northeast | 0.68 (0.42~1.10) | 0.115 |
| **Region (rural vs urban)** | 0.81 (0.65~1.01) | 0.064 |
| **Grade (vs 1)** |  |  |
| 2 | 0.83 (0.61~1.14) | 0.244 |
| 3 | 0.68 (0.50~0.93) | 0.017 |
| 4 or 5 | 0.63 (0.44~0.91) | 0.014 |
| Postgraduate | 0.37 (0.21~0.66) | <0.001 |
| **Exercise frequency (vs <once a week)** |  |  |
| 1~2 times a week | 0.91 (0.72~1.16) | 0.469 |
| ≥3 times a week | 0.74 (0.56~0.98) | 0.034 |
| **Social relations** |  |  |
| **Childhood bullying experience (vs never)** |  |  |
| Seldom | 1.34 (1.08~1.68) | 0.009 |
| Sometimes | 3.16 (2.28~4.39) | <0.001 |
| Often | 4.97 (2.45~10.08) | <0.001 |
| **Family Information** |  |  |
| **Parenting style (vs authoritative)** |  |  |
| Authoritarian | 1.10 (0.81~1.47) | 0.549 |
| Neglectful | 0.97 (0.74~1.26) | 0.796 |
| Permissive | 2.48 (1.25~4.93) | 0.009 |
| **Number of siblings (vs 0)** |  |  |
| 1 | 1.39 (1.12~1.73) | 0.003 |
| 2 | 2.61 (1.92~3.54) | <0.001 |
| ≥3 | 1.15 (0.66~2.00) | 0.634 |
| **Childhood adversity experience (vs never)** |  |  |
| Seldom | 1.68 (1.33~2.12) | <0.001 |
| Sometimes | 2.46 (1.82~3.32) | <0.001 |
| Often | 7.76 (4.17~14.45) | <0.001 |
| **Self-evaluation** |  |  |
| **Mobile phone dependence (vs no)** |  |  |
| General | 2.32 (1.82~2.96) | <0.001 |
| Yes | 4.40 (3.31~5.84) | <0.001 |
| **Appearance satisfaction (vs not very satisfied)** | |  |
| Moderately satisfied | 0.85 (0.64~1.12) | 0.248 |
| Quite satisfied | 0.67 (0.49~0.91) | 0.011 |
| **Mental health self-assessment (bs not very healthy)** | |  |
| Moderately healthy | 0.91 (0.58~1.41) | 0.661 |
| Quite healthy | 0.42 (0.27~0.66) | <0.001 |

**Supplementary Table S1.** Ordinal logistic regression for the outcome variable divided by tertiles (sensitivity analysis). The outcome variable was divided by tertiles into 3 groups of low score (0~5, n=637), middle score (6~17, n=624), and high score (18~48, n=598). McFadden's R²=0.208. GVIF for all variables is less than 1.57. OR, odds ratio; CI, confidence interval.

| **Factor** | **OR (95%CI)** | ***P* Value** |
| --- | --- | --- |
| **Demographic information** |  |  |
| **Gender (female vs male)** | 0.70 (0.59~0.84) | <0.001 |
| **Region (rural vs urban)** | 0.76 (0.62~0.93) | 0.007 |
| **Grade (vs 1)** |  |  |
| 2 | 0.81 (0.60~1.08) | 0.153 |
| 3 | 0.76 (0.56~1.01) | 0.060 |
| 4 or 5 | 0.65 (0.46~0.91) | 0.012 |
| Postgraduate | 0.39 (0.23~0.66) | <0.001 |
| **Exercise frequency (vs <once a week)** |  |  |
| 1~2 times a week | 1.12 (0.89~1.40) | 0.329 |
| ≥3 times a week | 0.78 (0.60~1.01) | 0.061 |
| **Social relations** |  |  |
| **Childhood bullying experience (vs never)** |  |  |
| Seldom | 1.27 (1.03~1.57) | 0.024 |
| Sometimes | 3.07 (2.26~4.17) | <0.001 |
| Often | 4.02 (2.17~7.42) | <0.001 |
| **Family Information** |  |  |
| **Parenting style (vs authoritative)** |  |  |
| Authoritarian | 0.95 (0.72~1.26) | 0.729 |
| Neglectful | 1.04 (0.81~1.33) | 0.760 |
| Permissive | 2.37 (1.26~4.45) | 0.007 |
| **Number of siblings (vs 0)** |  |  |
| 1 | 1.32 (1.08~1.61) | 0.007 |
| 2 | 2.42 (1.82~3.22) | <0.001 |
| ≥3 | 1.33 (0.79~2.23) | 0.286 |
| **Childhood adversity experience (vs never)** |  |  |
| Seldom | 1.63 (1.30~2.03) | <0.001 |
| Sometimes | 2.39 (1.80~3.17) | <0.001 |
| Often | 8.39 (4.80~14.68) | <0.001 |
| **Self-evaluation** |  |  |
| **Mobile phone dependence (vs no)** |  |  |
| General | 2.20 (1.76~2.76) | <0.001 |
| Yes | 4.49 (3.44~5.86) | <0.001 |
| **Appearance satisfaction (vs not very satisfied)** | |  |
| Moderately satisfied | 0.79 (0.61~1.03) | 0.083 |
| Quite satisfied | 0.68 (0.50~0.91) | 0.009 |
| **Mental health self-assessment (bs not very healthy)** | |  |
| Moderately healthy | 1.00 (0.67~1.50) | 0.988 |
| Quite healthy | 0.49 (0.33~0.75) | <0.001 |

**Supplementary Table S2**. Ordinal logistic regression for the outcome variable divided by quartiles (sensitivity analysis). The outcome variable was divided by quartiles into 4 groups of very low score (0~3, n=478), low score (4~10, n=478), high score (11~21, n=441), and very high score (22~48, n=462). McFadden's R²=0.171. GVIF for all variables is less than 1.60. OR, odds ratio; CI, confidence interval.

| **Subjective feelings of COVID-19's preventive measures on social phobia** | | **Total, n(%)** | **Change of social phobia score in pre- and early-COVID-19 periods** | | |
| --- | --- | --- | --- | --- | --- |
|  |  |  | **Reduced, n(%)** | **Unchanged, n(%)** | **Increased, n(%)** |
| **Feelings of wearing a mask on social phobia** | | |  |  |  |
|  | Alleviated | 863 (46.4) | 265 (49.5) | 206 (40.2) | 392 (48.3) |
|  | Unchanged | 808 (43.5) | 200 (37.4) | 279 (54.5) | 329 (40.5) |
|  | Aggravated | 188 (10.1) | 70 (13.1) | 27 (5.3) | 91 (11.2) |
| **Feelings of setting a baffle on the canteen dining table on social phobia** | | | |  |  |
|  | Alleviated | 766 (41.2) | 247 (46.2) | 177 (34.6) | 342 (42.1) |
|  | Unchanged | 943 (50.7) | 229 (42.8) | 312 (60.9) | 402 (49.5) |
|  | Aggravated | 150 (8.1) | 59 (11.0) | 23 (4.5) | 68 (8.4) |
| **Feelings of keeping a one-meter social distance on social phobia** | | | |  |  |
|  | Alleviated | 744 (40.0) | 228 (42.6) | 182 (35.5) | 334 (41.1) |
|  | Unchanged | 882 (47.4) | 224 (41.9) | 286 (55.9) | 372 (45.8) |
|  | Aggravated | 233 (12.5) | 83 (15.5) | 44 (8.6) | 106 (13.1) |
| **Feelings of vaccination against COVID-19 on social phobia** | | | |  |  |
|  | Alleviated | 616 (33.1) | 163 (30.5) | 158 (30.9) | 295 (36.3) |
|  | Unchanged | 1101 (59.2) | 318 (59.4) | 332 (64.8) | 451 (55.5) |
|  | Aggravated | 142 (7.6) | 54 (10.1) | 22 (4.3) | 66 (8.1) |
| **Feelings of online presentation on social phobia** | | |  |  |  |
|  | Alleviated | 768 (41.3) | 205 (38.3) | 207 (40.4) | 356 (43.8) |
|  | Unchanged | 904 (48.6) | 257 (48.0) | 271 (52.9) | 376 (46.3) |
|  | Aggravated | 187 (10.1) | 73 (13.6) | 34 (6.6) | 80 (9.9) |
| **Feelings of online asking or answering questions on social phobia** | | | |  |  |
|  | Alleviated | 816 (43.9) | 229 (42.8) | 221 (43.2) | 366 (45.1) |
|  | Unchanged | 817 (43.9) | 217 (40.6) | 253 (49.4) | 347 (42.7) |
|  | Aggravated | 226 (12.2) | 89 (16.6) | 38 (7.4) | 99 (12.2) |

**Supplementary Table S3**. Subjective feelings of COVID-19’s preventive measures on social phobia (n=1859).

| **Factor** | | **OR (95%CI)** | ***P* value** |
| --- | --- | --- | --- |
| **Subjective feelings of COVID-19's preventive measures on social phobia** | | | |
| **Feelings of setting a baffle on the canteen dining table on social phobia (vs alleviated)** | | | |
|  | Unchanged | 1.30 (1.07,1.58) | 0.008 |
|  | Aggravated | 1.11 (0.77,1.61) | 0.578 |
| **Feelings of getting vaccinated on social phobia (vs alleviated)** | | |  |
|  | Unchanged | 0.73 (0.59,0.89) | 0.002 |
|  | Aggravated | 0.78 (0.53,1.15) | 0.212 |
| **Covariant** | |  |  |
| **Region (rural vs urban)** | | 0.85 (0.69,1.03) | 0.102 |
| **BMI (vs normal)** | |  |  |
|  | Overweight | 1.14 (0.85,1.51) | 0.387 |
|  | Obesity | 1.78 (1.05,3.01) | 0.033 |
|  | Underweight | 1.15 (0.93,1.42) | 0.190 |
| **Exercise frequency (vs <once a week)** | |  |  |
| 1~2 times a week | | 0.80 (0.64,1.00) | 0.046 |
| ≥3 times a week | | 0.85 (0.66,1.08) | 0.181 |
| **Never participate in student organizations (vs ever)** | | 1.17 (0.96,1.44) | 0.120 |
| **Childhood bullying experience (vs never)** | |  |  |
| Seldom | | 1.09 (0.90,1.32) | 0.372 |
| Sometimes | | 0.79 (0.61,1.02) | 0.069 |
| Often | | 0.66 (0.40,1.10) | 0.113 |
| **Number of siblings (vs 0)** | |  |  |
|  | 1 | 1.10 (0.91,1.34) | 0.331 |
|  | 2 | 1.38 (1.04,1.83) | 0.025 |
|  | ≥3 | 1.62 (0.96,2.73) | 0.071 |

**Supplementary Table S4**. Ordinal logistic regression of determinants of retrospective self-reported change in college students’ social phobia in pre- and early-COVID-19 periods. McFadden's R²=0.011. GVIF for all variables is less than 1.40. OR, odds ratio; CI, confidence interval; COVID-19, coronavirus disease 2019; BMI, body mass index.
